# Application of the “Plan-Do-Check-Action” plan in improving the pass rate of the “National Medical Licensing Examination”

**DOI:** 10.1186/s12909-024-05706-6

**Published:** 2024-07-02

**Authors:** Shu Zhou, Xian Zhang, Hao Zhang, Donglei Zhang, Renxiong Wei, Miao Yang

**Affiliations:** 1 Department of Hematology, Zhongnan Hospital, Wuhan University, Wuhan, Hubei 430071 China; 2 Teaching Affair Office, Zhongnan Hospital of Wuhan University, Wuhan, Hubei 430071 China; 3 Department of Spine and Bone Oncology, Zhongnan Hospital of Wuhan University, Wuhan, Hubei 430071 China; 4 Department of Cardiology, The Second Affiliated Hospital of Tianjin Medical University, Tianjin, 300000 China

**Keywords:** PDCA plan, National Medical Licensing Examination, Pass rate, Education

## Abstract

**Background:**

The National Medical Licensing Examination (NMLE) is the only objective, standardized metric to evaluate whether a medical student possessing the professional knowledge and skills necessary to work as a physician. However, the overall pass rate of NMLE in our hospital in 2021 was much lower than that of Peking Union Medical College Hospital, which was required to be further improved.

**Methods:**

To find the reasons for the unsatisfactory performance in 2021, the quality improvement team (QIT) organized regular face-to-face meetings for in-depth discussion and questionnaire, and analyzed the data by “Plato analysis” and “Brainstorming method”. After finding out the reasons, the “Plan-Do-Check-Action” (PDCA) cycle was continued to identify and solve problems, which included the formulation and implementation of specific training plans by creating the “Gantt charts”, the check of effects, and continuous improvements from 2021 to 2022. Detailed information about the performance of students in 2021 and 2022, and the attendance, assessment, evaluation and suggestions from our hospital were provided by the relevant departments, and the pass rate-associated data was collected online.

**Results:**

After the PDCA plan, the pass rate of NMLE in our hospital increased by 10.89% from 80.15% in 2021 to 91.04% in 2022 (*P* = 0.0109), with the pass rate of skill examination from 95.59% in 2021 to 99.25% in 2022 (*P* = 0.0581) and theoretical examination from 84.5% in 2021 to 93.13% in 2022 (*P* = 0.027). Additionally, the mean scores of all examinees increased with the theoretical examination score increasing from 377.0 ± 98.76 in 2021 to 407.6 ± 71.94 in 2022 (*P* = 0.004).

**Conclusions:**

Our results showed a success application of the PDCA plan in our hospital which improved the pass rate of the NMLE in 2022, and the PDCA plan may provide a practical framework for future medical education and further improve the pass rate of NMLE in the next year.

## Introduction

The National Medical Licensing Examination (NMLE) is an entry examination developed in accordance with the Law of the People’s Republic of China on Practicing Physicians to evaluate whether an applicant for physician qualification possesses the professional knowledge and skills necessary to work as a physician [1]. The “Opinions on Strengthening Medical Education Collaboration and Implementing Excellence in Doctor Education and Training Program 2.0” and other documents have pointed out that we should closely focus on the implementation of the Healthy China Strategy, deepen medical education collaboration, promote competency-oriented medical education and teaching reforms, and cultivate top-notch medical talents to serve the construction of a healthy China. Relevant policies emphasized that the pass rate of the NMLE should be taken as an important element in evaluating the quality of medical personnel training, and the enrollment of colleges and universities with a pass rate of the NMLE lower than 50% for three consecutive years should be reduced. The pass rate of the NMLE is an important standard to test and measure the teaching quality of clinical medicine in medical schools, and is of great significance for strengthening the construction of physician teams, improving the quality and level of medical and healthcare services, and safeguarding people’s health and life safety.

At our hospital, the pass rate of NMLE in 2021 was 80.15%. In contrast, clinical medicine students at Peking Union Medical College Hospital, the TOP1 medical college in China, passed with a pass rate of 95.08%. Although it is well-known that the clinical medical students of Peking Union Medical College Hospital may have relatively stronger learning abilities than the students in our medical school which ranking 13th in China in the “2020 Chinese Hospitals/Chinese Medical Schools Science and Technology Measurement (STEM)” ranking, the quality of our students is also at the upper end of the scale in china, and they should have done better in the NMLE. In any case we should hold the Peking Union medical college as a standard of progress, and the pass rate of the NMLE in our hospital still need to be further improved.

In recent years, “Plan-Do-Check-Action” (PDCA) procedure, a procedural, standardized, scientific quality management work method proposed by Dr. Deming, an American quality management expert, has been widely used in clinical teaching and management, such as the standardized training of resident physicians and nursing management [2–5]. In order to improve the clinical competency and the pass rate of the NMLE for medical residents, we started to adopt the PDCA procedure in 2021 to deeply analyze the problems affecting the pass rate of the NMLE, comprehensively manage the whole process of the activity, and formulate a scientific and effective work plan for the continuous improvement. To evaluate the efficiency of PDCA plan, we compared the pass rate of the NMLE in 2021 and 2022 and analyzed the scores from the aspect of various subjects. The pass rate of the NMLE is a quantitative indicator of the performance of the PDCA training program.

## Methods

### Information Collection

All the examinees who took the NMLE in our hospital from 2021 to 2022 were taken as the research subjects, and they were divided into an observation group and a control group according to whether they accepted the PDCA plan. The participants in the observation group accepted the PDCA plan and took the NMLE in 2022, while the participants in the control group did not accept the PDCA plan and took the NMLE in 2021. Between the two groups, there was no statistically significant variation in the age or gender distribution. Detailed information about the performance of students, and the attendance, assessment, evaluation and suggestions from our hospital were provided by the relevant departments, and the pass rate-associated data was collected online.

### The PDCA Plan

**Fig. 1:**
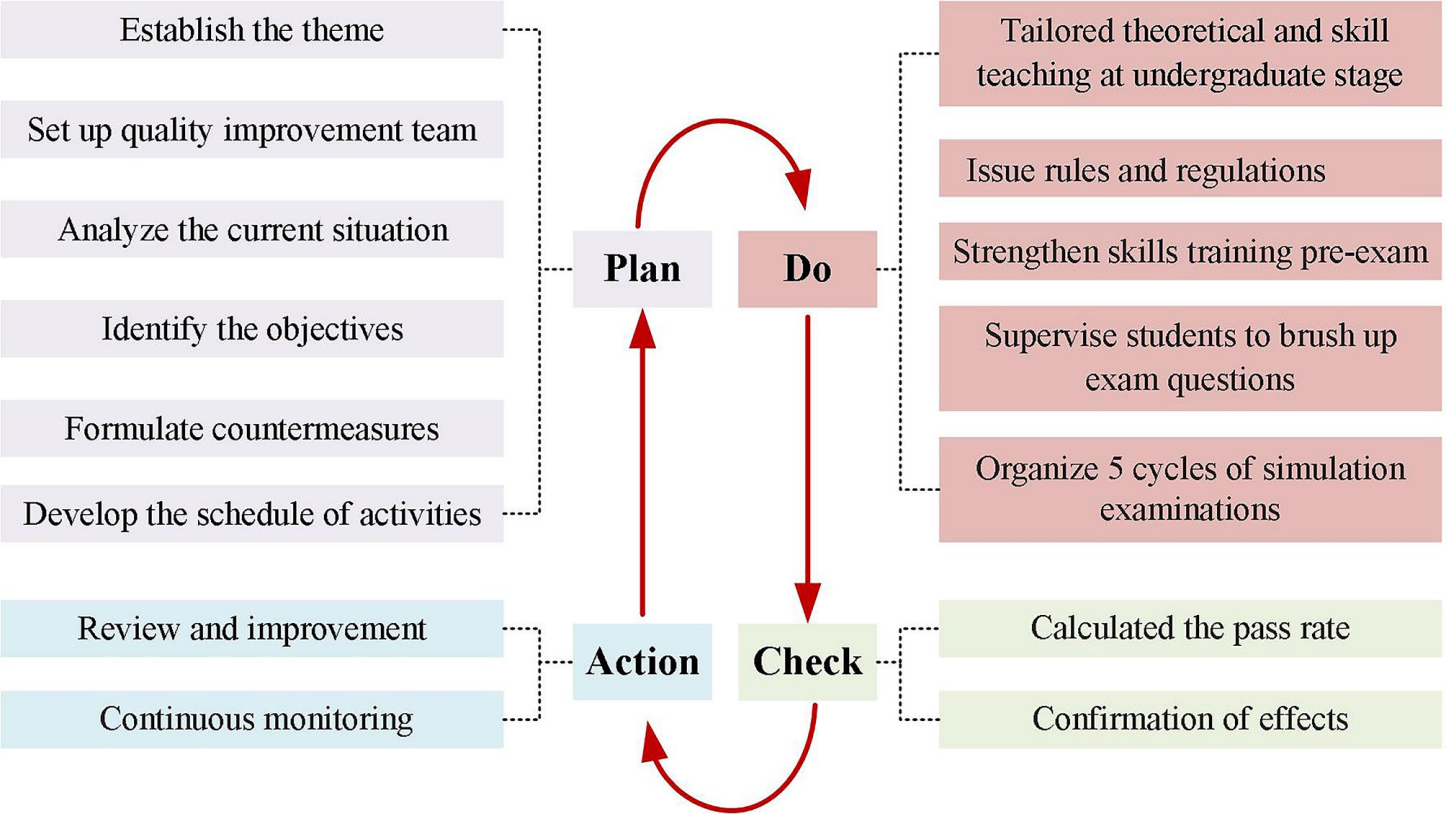
The PDCA Plan

We implemented the PDCA strategy, which had four stages, including Plan, Do, Check, and Action (Fig. 1), to improve the pass rate of NMLE in our hospital in 2022. The detailed operating procedures are exhibited as below.

### Plan

**Fig. 2:**
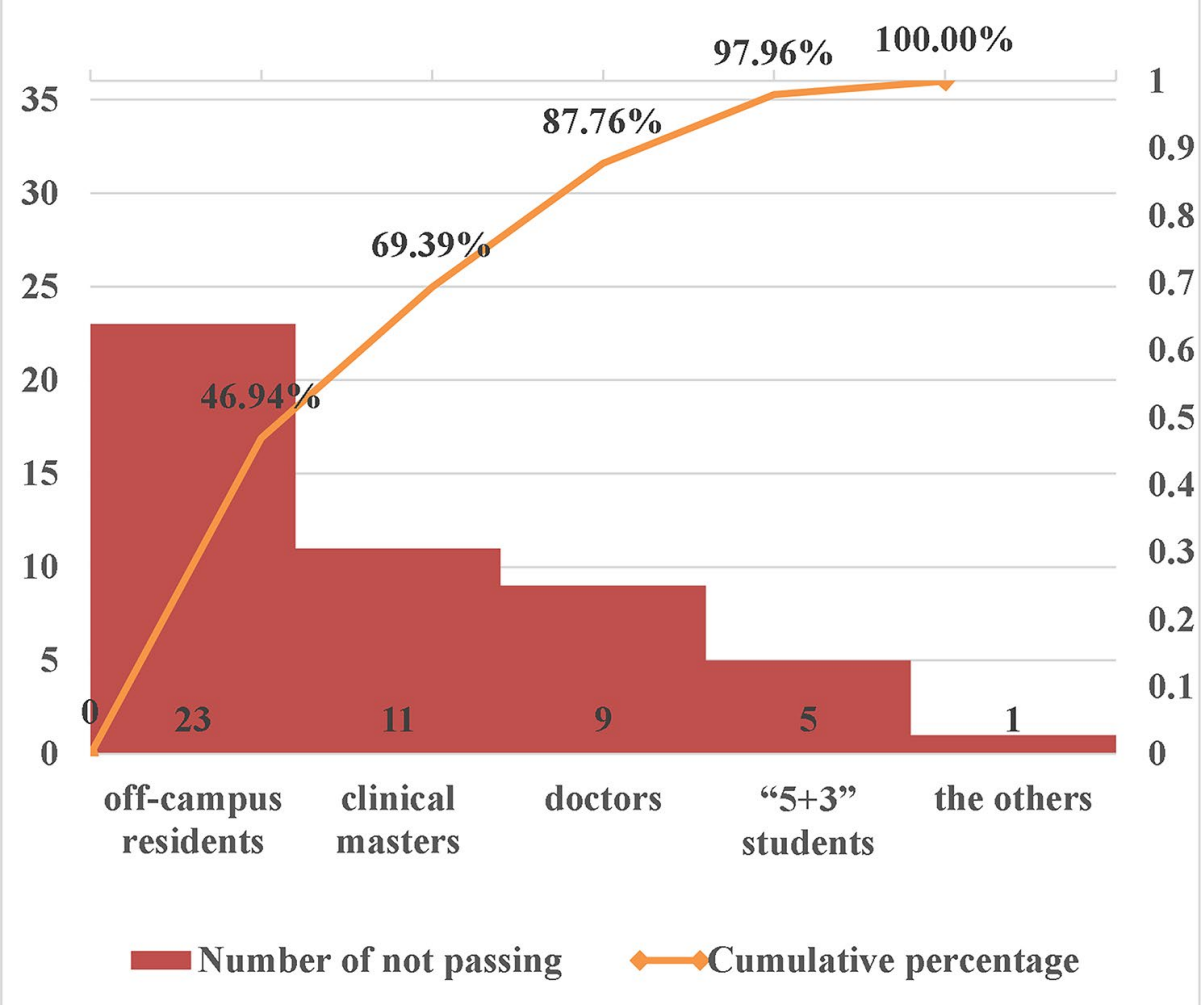
The “Plato analysis” of the poor performance in 2021. The Plato analysis showed that the off-campus residents, the clinical masters (excluding “5 + 3” students), and the doctors occupied a major proportion

**Fig. 3:**
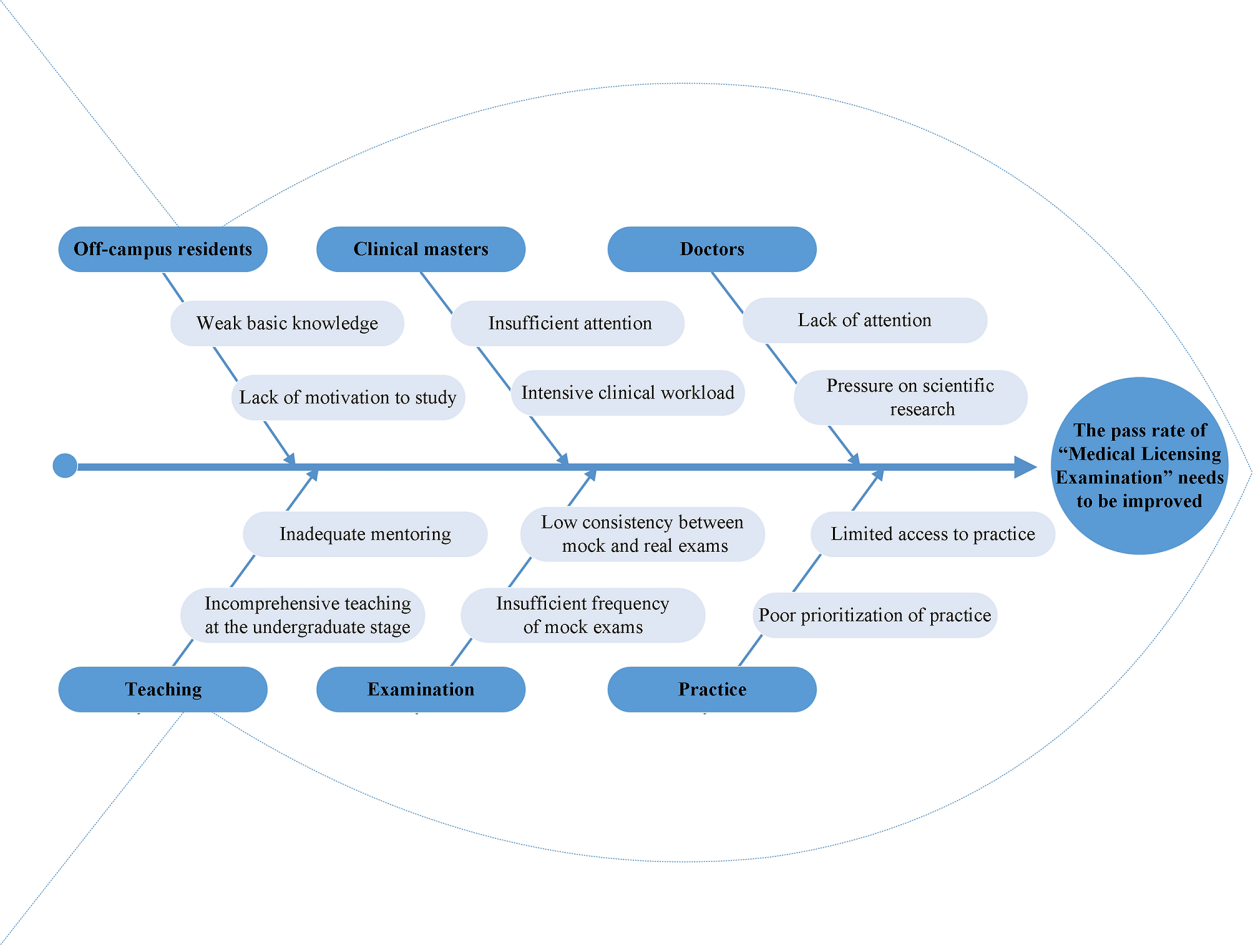
The “fishbone diagram” of the poor performance in 2021. The brainstorming method was utilized to analyze the factors that contributing to the poor performance in 2021 from the perspective of personnel categories

**Fig. 4:**
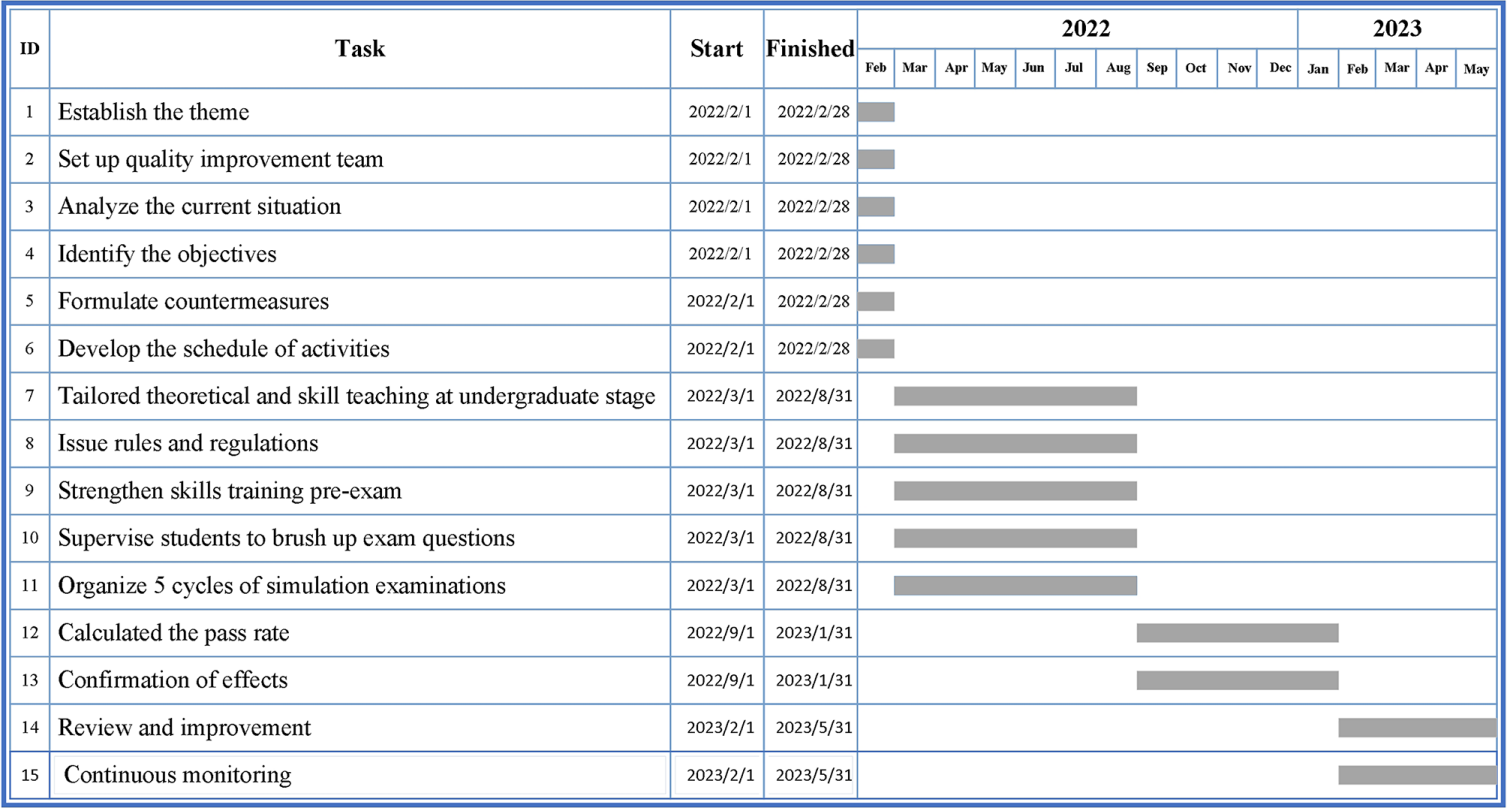
The PDCA schedule developed by the “Gantt chart”

A quality improvement team (QIT) composed of the medical education and hospital departments was set up. 18 participants were joined in the QIT with the age of 29–55 years old, and half of them came from the department of medical education. The work of the QIT in the “Plan” stage included: (1) Established the theme: improving the pass rate of NMLE in our hospital. (2) Analyzed the current situation. According to the different origins of personnel, the examinees in NMLE were divided into five categories, including the off-campus resident physicians who graduated from other medical schools, the clinical masters who would graduate with four certificates including the certificate of standardized resident training, the “5 + 3” students who just finished the 5 years of bachelor of science degree training in clinical medicine and would continuously receive 3 years of standardized resident training in our hospital, the doctors at sixth-year of an “eight-year doctorate in clinical medicine” or the PhD newly entering our hospital to work and the others. To dig out the factors that contributed to the poor performance in 2021, we organized conferences for faculty and students and questionnaire research to gain a comprehensive and in-depth understanding of the problems of various categories of personnel in the NMLE, and we also analyzed the data through “Plato analysis” (Fig. 2) and “Brainstorming method” (Fig. 3). (3) Identified the objectives: focusing on talent development, clinical competency, and improving the pass rate of the NMLE. (4) Formulated countermeasures and tailored training plans. (5) Developed the schedule of activities and arrange theoretical studies and training assessments by using the method of “Gantt chart” (Fig. 4). Subsequently, the QIT analyzed the results of the examinees with an emphasis on the pass rate of the NMLE before making further plans. Constant adjustments in this step were taken according the monthly QIT meeting until a complete and practical solution was determined.

### Do

When the plan has been made, the next stage was to implement (Do). We constantly insist on talent training as the first step, clinical competence as the primary benchmark, and increasing NMLE pass rates as the end goal; as a result, all phases, from the study of fundamental medical knowledge to the development of clinical reasoning to exam preparation, should be strongly emphasized and supported. The undergraduate stage is the foundation stage for medical students to grasp medical professional knowledge, even if the application period for the examination is after undergraduate stage and the examinees are already in the standardized residency training or postgraduate stage. Therefore, we adopt a two-pronged approach with theoretical teaching and practical teaching, and carried out the study of the content of the NMLE throughout the whole process from “teaching” to “examination”, so as to consolidate the basic knowledge of medicine for the medical students and make a scientific plan for the path of growth. The enhancement program incorporated two stages, including the “undergraduate stage” and the “pre-examination stage”. Under the “undergraduate stage” of the enhancement program, the content of the theoretical, practical teaching and the evaluation of learning effects were designed according to requirements of the NMLE. Under the “pre-examination stage” of the enhancement program, the QIT held monthly working meetings to promote multiple rounds of simulation examinations for medical practitioners, strengthen the training of weak links, supervise students to brush up on the questions and investigate the situation of the students.

When determining the main reasons of the poor performance in 2021, the QIT strategically attempted to address these issues by taking a number of steps, such as increasing examinees’ attention, providing training venues and individualized guidance, supervising students to review exam questions and organizing simulation examinations; and creating “Gantt charts” to help implementing these measures. In order to increase the attention of examinees and supervisors, the QIT issued rules and regulations for both examinees and supervisors. For example, the pass rate of examinees was factored into the supervisors’ promotions, award applications, and annual evaluations by the department director, which helped to stimulate the enthusiasm of both the trainees and the supervisors. To improve the skill ability of examinees, the Training Center provided more specialized training venues, such as “standardized patient platforms” with a wide variety of clinical cases for “Medical History Collection,” dummies for “Physical Examination” and “Specialized Skills”, and extended training time to increase the number of practice opportunities for examinees who were engaged in clinical or research work. In addition, the skill courses in relation to electrocardiogram and X-ray interpretation and others were developed by the senior doctors from the corresponding department, and the students were required to participate with attendance records. Moreover, examinees who had problems in skill operation could ask for individual guidance from supervisors.

The theoretical subjects of the NMLE are divided into four parts, including basic classification, humanities classification, preventive medicine classification and clinical classification, which comprehensively evaluate the overall mastery of medical knowledge and their ability to internalize the basic knowledge of medicine into the diagnosis and treatment of diseases. To comprehensively acquire the theoretical knowledge, the QIT supervised students to brush up on exam questions from February 2022, and an attendance record system was introduced to make comprehensive records to track the training process of trainees. The results were required to be feedback to the mentorship. Furthermore, the QIT organized 5 cycles of simulation examinations to test all examinees’ abilities monthly prior to the final examination. After obtaining the results of the simulation tests, the QIT conducted a careful analysis of the examinees’ performances, and those who did not pass the exam would receive feedback from supervisors before being re-evaluated. Moreover, the QIT held a symposium for them through face-to-face talks to figure out the difficulties, and informed the department to pay close attention to them.

### Check

In this step, the QIT calculated the pass rate for the next year to demonstrate the efficacy of the PDCA plan according to the comparisons with the results of previous years. Additionally, face-to-face symposiums and questionnaire studies were conducted to identify any issues and offer solutions. For example, in the questionnaire interview after the 2022 NMLE, 91% of the examinees believed that the PDCA management was helpful in their preparation for the NMLE while the others thought it ineffective. 14% of the examinees expressed that the amount of time given to prepare for the NMLE was not enough. They found it challenging to balance their time and energy due to the heavy clinical workload and demanding academic studies. Therefore, it is necessary to create effective time management strategies and delegate tasks based on the specific needs. Attendance, assessment, evaluation, and suggestion data were compiled to make necessary changes to the curriculum design and teaching strategies while also improving the quality of teaching.

### Action

The last step was “Action,” which primarily included review and improvements for standardization workflow and continuous monitoring. In this stage, it was proposed to review the whole process of the PDCA plan and formulate the standardized preparation program for the NMLE according to the national requirements and standards. Continuous monitoring was associated with prolonging the PDCA plan’s duration and carrying on with data collection for the following cycle.

### Statistical analysis

Statistical analyses were performed using Pearson’s chi-squared test, Yates-corrected chi-square test, Welch’s t test (Graphpad Prism 10) or Mann-Whitney U test (R software, version 4.2.1, Vienna, Austria). A P-value level of < 0.05 was considered significant.

## Results

### The pass rate of NMLE in 2021 and analysis of poor performance

In 2021, the total pass rate of NMLE in our hospital was 80.15%, which was much lower than that of clinical medicine students in Peking Union Medical College Hospital (95.08%). To analyze all the ones who did not pass the NMLE in our hospital in 2021, we conducted a Plato analysis (Fig. 2) which showed that the off-campus residents, the clinical masters (excluding “5 + 3” students), and the doctors occupied a major proportion, thus increasing their pass rate would be the key point to improving the overall pass rate in our hospital.

To further analyze the factors from the perspective of personnel categories, we use the brainstorming method to finally come up with the following conclusions (Fig. 3). First, the vast majority of off-campus resident physicians and the clinical masters (excluding “5 + 3” students) completed their undergraduate stage of education in other universities, and the main reasons for the poor pass rate of them are the weakness of basic knowledge and insufficient attention. Secondly, the doctors in their sixth year of an “eight-year doctorate in clinical medicine” or the scientific research doctors who have recently begun working at our hospital are highly skilled medical professionals and should have achieved a higher pass rate to reach the leading level. However, based on the current results, there was still some room for improvement; the main reasons for this were the lack of focus and the high workload related to clinical work and scientific research. Furthermore, starting from the growth of medical students, we analyzed the problems of teaching, examination, and students’ practice, and delved deeply into the reasons affecting the pass rate of the NMLE, such as incomplete instructions at the undergraduate stage, insufficient frequency of simulation examinations, and poor prioritization of practice, including inadequate undergraduate instruction, insufficient simulation exams, and a lackluster practice schedule. And the detailed conclusion has been exhibited in the fishbone diagram (Fig. 3).

### The pass rate of NMLE in 2022 and improvements

**Fig. 5:**
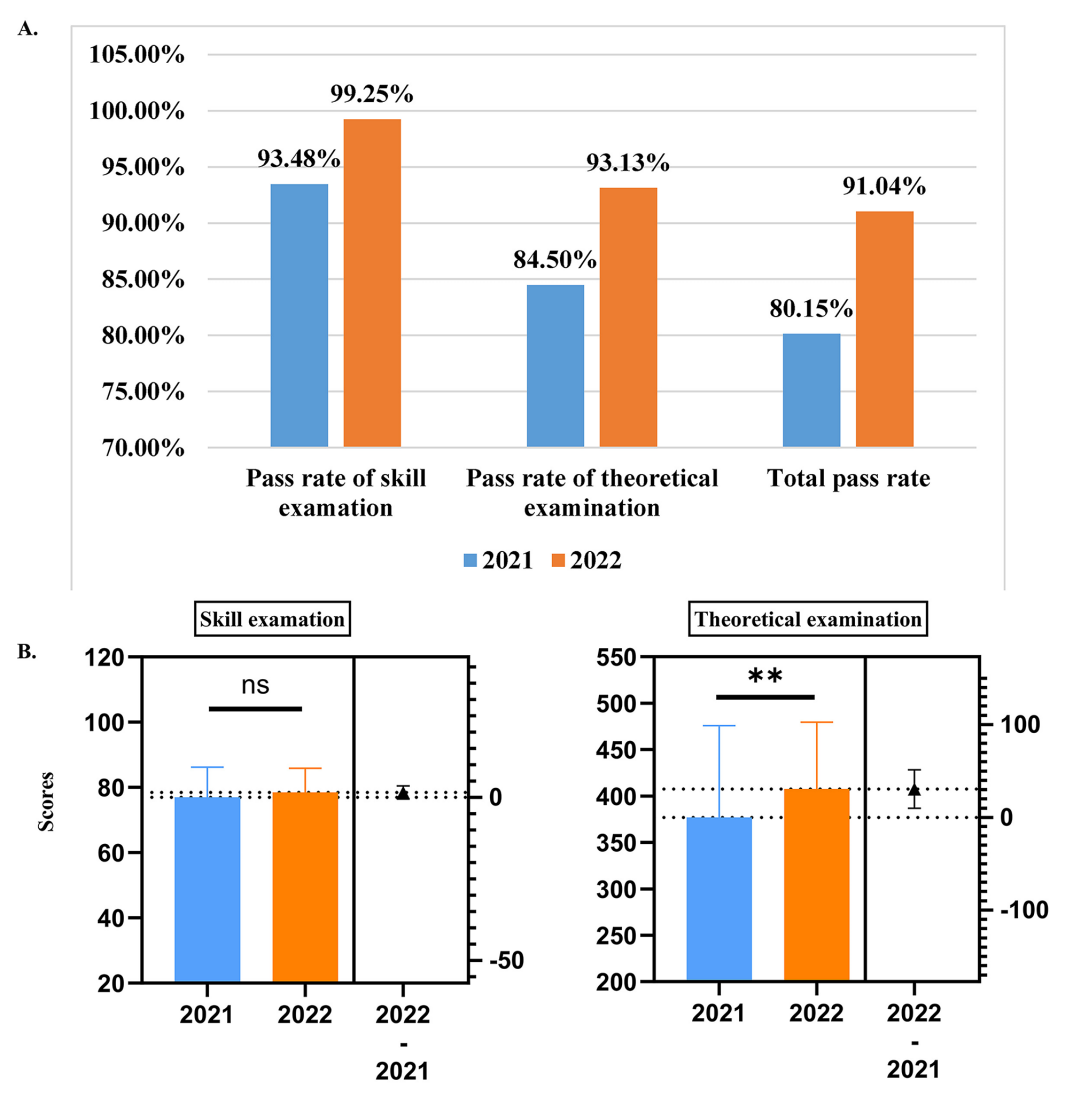
The performance of NMLE in 2021 and 2022

In 2022, the pass rate of NMLE in our hospital increased by 10.89% from 80.15% in 2021 to 91.04% in 2022 (*P* = 0.0109) with the pass rate of skill examination from 95.59% in 2021 to 99.25% in 2022 (*P* = 0.0581) and theoretical examination from 84.5% in 2021 to 93.13% in 2022 (*P* = 0.027) (Fig. 5A). In addition, we analyzed the scores of all examinees by comparing the mean ± SD of 2021 and 2022, and found that the score of the skill examination increased from 76.98 ± 9.187 in 2021 to 78.46 ± 7.444 in 2022, and the score of the theoretical examination increased from 377.0 ± 98.76 in 2021 to 407.6 ± 71.94 in 2022 (*P* = 0.004) (Fig. 5B). To further analyze the four parts of the theoretical examination, we found that the pass rate of humanities classification, preventive medicine classification and clinical classification significantly increased with statistical significance, but the pass rate of the basic medicine classification decreased in 2022 compared with that of 2021 (Table 1).

**Table 1:** The pass rate associated with subjects in 2021 and 2022

Subjects		Pass rate in 2021 *n* (%)	Pass rate in 2022 *n* (%)	χ 2	*P*-value
**Skill examination**		130 (95.59%)	133(99.25%)	3.591	0.0581
**Theoretical examination**		109 (84.5%)	122 (93.13%)	4.889	**0.027**
	**Basic**	83(64.34%)	38(29.01%)	32.61	< 0.0001
	**Humanities**	108(83.72%)	127(96.95%)	13.08	**0.0003**
	**Preventive**	116(89.92%)	130(99.24%)	11.07	**0.0009**
	**Clinical**	105(81.40%)	120(91.06%)	5.814	**0.0159**
**Total**		109 (80.15%)	122 (91.04%)	6.486	**0.0109**

In order to know more about the pass rate of detailed subjects among each category of examinees, the examinee scores for “5-year” undergraduates, “5 + 3” masters, and “8-year” doctors were examined independently. Among “5-year” undergraduates, the pass rate of skill examination in 2022 was not increased, while the pass rate of theoretical examination was increased even though without statistical significance. When analyzing each subject of theoretical examination, we found that the pass rate of pharmacology, morality and gynecology was significantly increased with statistical significance while the pass rate of pathology, microbiology, immunology and psychology was decreased. Among “5 + 3” masters, the pass rate of skill examination and theoretical examination in 2022 were both significantly increased, and the pass rate of morality and surgery was increased with statistical significance, while the pass rate of pathology, microbiology, immunology and psychology was also decreased. Among “8-year” doctors, the pass rate of skill examination in 2022 and 2021 was both 100%, and the pass rate of theoretical examination was increased even though without statistical significance, and the pass rate of physiology, regulations, morality, surgery and gynecology was significantly increased with statistical significance while the pass rate of pathology, microbiology, immunology and psychology was also decreased (Table 2). Overall, the results demonstrated the efficacy of the PDCA plan in improving the integrated pass rate of the NMLE.

**Table 2:** The pass rate of detailed subjects among each category of examinees in 2021 and 2022

Subjects		“5-year” undergraduates			“5 + 3” masters			“8-year” doctors		
		2021 *n* (%)	2022 *n* (%)	*P*	2021 *n* (%)	2022 *n* (%)	*P*	2021 *n* (%)	2022 *n* (%)	*P*
**Skill examination**		43 (93.5)	15 (93.8)	1	37 (92.5)	70 (100)	**0.046**	50 (100.0)	48 (100.0)	1
**Theoretical examination**		31 (67.4)	12 (75.0)	0.755	35 (87.5)	66 (94.3)	0.281	43 (86.0)	44 (91.7)	0.57
	Biochemistry	28 (60.9)	8 (50.0)	0.559	23 (57.5)	44 (62.9)	0.685	32 (64.0)	38 (79.2)	0.15
	Physiology	25 (54.3)	9 (56.2)	1	27 (67.5)	40 (57.1)	0.316	22 (44.0)	32 (66.7)	**0.04**
	Pathology	36 (78.3)	5 (31.2)	**0.001**	30 (75.0)	19 (27.1)	**< 0.001**	42 (84.0)	12 (25.0)	**< 0.001**
	Microbiology	22 (47.8)	1 (6.2)	**0.003**	26 (65.0)	12 (17.1)	**< 0.001**	33 (66.0)	8 (16.7)	**< 0.001**
	Immunology	27 (58.7)	1 (6.2)	**< 0.001**	30 (75.0)	2 (2.9)	**< 0.001**	43 (86.0)	6 (12.5)	**< 0.001**
	Pharmacology	18 (39.1)	11 (68.8)	**0.048**	18 (45.0)	43 (61.4)	0.113	30 (60.0)	31 (64.6)	0.795
	Pathophysiology	15 (32.6)	4 (25.0)	0.755	16 (40.0)	16 (22.9)	0.08	17 (34.0)	9 (18.8)	0.139
	Anatomy	5 (10.9)	2 (12.5)	1	6 (15.0)	15 (21.4)	0.46	7 (14.0)	4 (8.3)	0.57
	Regulations	19 (41.3)	9 (56.2)	0.386	31 (77.5)	63 (90.0)	0.094	28 (56.0)	43 (89.6)	**< 0.001**
	Morality	24 (52.2)	14 (87.5)	**0.017**	24 (60.0)	68 (97.1)	**< 0.001**	29 (58.0)	48 (100.0)	**< 0.001**
	Psychology	40 (87.0)	5 (31.2)	**< 0.001**	36 (90.0)	28 (40.0)	**< 0.001**	49 (98.0)	23 (47.9)	**< 0.001**
	Internal medicine	32 (69.6)	11 (68.8)	1	34 (85.0)	62 (88.6)	0.57	37 (74.0)	42 (87.5)	0.151
	Surgery	33 (71.7)	12 (75.0)	1	32 (80.0)	68 (97.1)	**0.004**	35 (70.0)	44 (91.7)	**0.014**
	Gynecology	26 (56.5)	14 (87.5)	**0.034**	32 (80.0)	65 (92.9)	0.064	37 (74.0)	46 (95.8)	**0.007**
	Pediatric	34 (73.9)	12 (75.0)	1	34 (85.0)	64 (91.4)	0.348	42 (84.0)	44 (91.7)	0.396

## Discussion

At present in China, NMLE performance is the only objective, standardized metric reported to evaluate whether an applicant for physician qualification possessing the professional knowledge and skills necessary to perform the medical work, thus improving the performance of NMLE is a priority for medical educators and students. However, the overall pass rate of NMLE in 2021 was much lower than that of Peking Union Medical College Hospital. In order to find the reasons for the unsatisfactory performance, the QIT took a series of measures, such as organizing regular face-to-face meetings for in-depth discussion and questionnaire research to find the reasons for the unsatisfactory performance. Through analyzing the data by “Plato analysis” (Fig. 2) and “Brainstorming method” (Fig. 3), we found that the primary factors that could be corrected were the weakness of basic knowledge, deficiency of attention, time constraints due to intensive clinical work and scientific research occupying the preparation time, as well as the lack of comprehensive teaching resources, sensible exercise plans, specialized training venues and regular simulation examinations. Analyzing the current situation, especially the failing reasons, is the critical step of the PDCA cycle. After finding out the reasons, we continued the PDCA cycle to identify and solve problems, and then to formulate the standardized preparation program for NMLE according to the national requirements and guidelines. Eventually, the importance of implementing a PDCA plan to increase the NMLE certification rate has been shown by the notable improvement in student assessment results. Furthermore, by adopting the PDCA cycle, our organization demonstrated commendable performance in the year 2022. Additionally, we gained a comprehensive understanding of the efficacy of teaching and learning in many disciplines by doing a thorough analysis of the specific subjects within each category of examinees. For example, the information in Tables 1 and 2 indicates that the teaching and learning methods of skill examinations, humanities and preventive subjects are worthwhile to keep on while the basic subjects, especially pathology, microbiology and immunology, are required to be improved. Therefore, these findings will serve as a valuable resource for informing instructional modifications and facilitating enhanced feedback provision in the subsequent academic year.

However, some conspicuous limitations are also in the study. The first one is the small sample size of our research. Due to data confidentiality and the lack of open database for NMLE examinations in China, we did not have access to the detailed results of residents’ performances at other hospitals. Therefore, all the data were derived from our own hospital. In the future, we hope that teaching hospitals can cooperate with others and strengthen data sharing to achieve better medical teaching performance. Another constraint is the absence of controllable variables. While it is undeniable that the pass rate of the NMLE in 2022 is higher than that of 2021, there are still some potential factors that could affect the conclusion, such as the influence of the COVID-19 epidemic, the potential differences in the difficulty of the basic medical questions and the diverse backgrounds of medical students. But in fact, during the three-year pandemic in China, all the students were isolated at schools and the situation of the COVID-19 epidemic at the university campus in 2021 and 2022 were comparable. Therefore, we believe that the impact on educational content and instruction in 2021 is also comparable to that in 2022. Nevertheless, to definitely eliminate the above confounding factors, we hope to develop a prospective study to demonstrate the efficiency of PDCA plan during the same period in the future. The third limitation is the lack of participation and satisfaction survey. Due to busy and uncontrollable work schedules, there were still some students, such as clinical masters and doctors who carried heavy clinical burdens and tough academic studies, having difficulty in balancing time and energy to perform well in all aspects. In the following year, we will formulate an individualized schedule so that more students can participate according to different scenarios and develop the satisfaction survey for better improvements.

Recently, the PDCA plan has been widely used in clinical teaching and management, such as the standardized training of resident physicians and nursing management, and exhibited excellent efficiency [2–5], but in aspect of NMLE, no study has been reported about the application of PDCA plan in promoting the pass rate of NMLE. In this study, we observed results from one year and saw an increase in the pass rate of the NMLE in 2022 after the application of the PDCA plan. We will continue to develop the PDCA cycle for NMLE and make it more objective and reliable, and we hope that our educational experience can bring benefits to other hospitals or areas of medical education.

## Conclusion

To improve the clinical competency and the pass rate of the NMLE for medical residents, we used a procedural, standardized, scientific quality management work method which is the PDCA plan. Although the sample size was small and the observation time was short, we witnessed the success of the PDCA plan through an increased pass rate in the NMLE. In spite of the improvements, there are still some limits, so we will continue to develop the PDCA plan for the NMLE in the next cycle and make it more objective and reliable, and we hope that our educational experience can bring benefits to other hospitals or areas of medical education.

## Data Availability

All data generated or analysed during this study are included in this published article.
